# Promote or inhibit? The effect of the whole chain development of intellectual property on manufacturing firm performance

**DOI:** 10.3389/fpsyg.2023.1100865

**Published:** 2023-02-23

**Authors:** Liyan Yu, Chengfan Zhao

**Affiliations:** School of Economics and Management, Heilongjiang University, Harbin, China

**Keywords:** intellectual property creation, intellectual property utilization, intellectual property protection, intellectual property management, intellectual property service, manufacturing firm performance, firm heterogeneity

## Abstract

With the promotion of intellectual property strategy, the whole chain development of intellectual property environment faced by manufacturing firms has been improved, but the effect of the whole chain development of intellectual property on firm performance still needs to be studied. The purpose of this study is to investigate the impact of the whole chain development of intellectual property on different types of manufacturing firm performance. A panel regression model is constructed based on listed manufacturing firms from 2010 to 2020, and this is tested empirically. The results of the study show that the whole chain development of intellectual property has a promotion effect on manufacturing firm performance, among which the promotion effect of intellectual property services is the strongest, followed by intellectual property utilization and intellectual property creation, and the promotion effect of intellectual property management has a significant lag, while the relationship between intellectual property protection and manufacturing firms performance is significantly negative. The promotion effect of the whole chain development of intellectual property on different types of manufacturing firm performance is heterogeneous, and the promotion effect of the whole chain development of intellectual property on the performance of small-scale, non-state-owned, medium-high-technology manufacturing firms is stronger than that of large-scale, state-owned, low-technology manufacturing firms. This study not only extends the research direction of intellectual property, but also adds to the literature on firm performance. In addition, the findings of this paper help government authorities to further optimize the strategic layout of the whole chain development of intellectual property, promote manufacturing firms to solve internal management problems, and achieve a positive interaction between the top-level design of the intellectual property and the bottom-level logic of manufacturing firms.

## 1. Introduction

The manufacturing industry is the foundation of the real economy, and promoting the high-quality development of manufacturing industry is an important part of building a modernized economic system. China attaches great importance to the development of the manufacturing industry, manufacturing value added has ranked first in the world for 12 consecutive years. In 2021, the total manufacturing industry in China reached $4.864 trillion, accounting for 30% of the global proportion. However, China’s manufacturing industry is still big but not strong. Weak independent innovation capability, high external dependence on core technologies, the high energy consumption of products, and high carbon emissions are still the biggest constraints to the high-quality development of the manufacturing industry (Yu and Hu, 2018). So policymakers focus on intellectual property rights (IPR) and regard the development of IPR as a policy tool to stimulate the vitality of manufacturing firms and promote the high-quality development of the manufacturing industry. In 2018, the Ministry of Industry and Information Technology of China formulated the “Manufacturing Intellectual Property Action Plan (2018–2020),” emphasizing that firms should be the main body to make up for the unbalanced and insufficient development of IPR in the manufacturing industry. Then in 2021, the State Council of China issued the “Outline for Building a Powerful Intellectual Property Country (2021–2035),” which proposed to realize the whole chain development of intellectual property (WCDIP), including intellectual property creation (IPC), intellectual property utilization (IPU), intellectual property protection (IPP), intellectual property management (IPM), intellectual property service (IPS), and optimize the IPR development system, and provide a solid guarantee for the development of various industries, including the manufacturing industry. In recent years, the IPR creation mechanism, market operation mechanism, protection system, regulatory system, and public service system faced by the manufacturing firms have been improved, but has the macro-level WCDIP promoted the performance of micro-manufacturing firms? A study by Wu and Tang (2016) found that the stronger the enforcement of IPP, the more the patent output of firms, and the more significant the promotion of patent output on firm performance. Using panel data from Korean firms, Cho et al. (2015) empirically tested that the intensity of IPP has different effects on different industries and firms, specifically stronger IPP is more favorable to patent-intensive industries, but is detrimental to small and medium-sized firms. Wang and Hu (2021) analyzed the mechanism of macro-institutional effects on micro-firms with a sample of startups and concluded that the IPP system has a positive effect on both firm innovation and performance.

In summary, scholars have conducted some studies on the relationship between IPR and firm performance, but the following shortcomings remain: First, the literature on the impact of macro-level IPR development on firm performance is scarce and the research is mostly limited to the IPP perspective, while few scholars have conducted comprehensive studies from the perspective of WCDIP. Second, the theoretical and empirical research on the heterogeneity effect at the firm level is relatively weak, especially the systematic research on the impact of intellectual property development on the performance of heterogeneous manufacturing firms. This paper explores the role and heterogeneity effect of WCDIP on manufacturing firm performance (MFP) through empirical research, hoping to provide a theoretical basis for the government to promote MFP through WCDIP, and to provide a reference for manufacturing firms to achieve high-quality development through matching the national policies on WCDIP. The marginal contributions of this paper are as follows: First, we use the entropy value to construct an evaluation index system for WCDIP in five dimensions of IPC, IPU, IPP, IPM, and IPS, which enriches the measurement method of WCDIP. Second, using the panel regression models, we explore the impact of WCDIP and the development of each dimension on MFP from the macro level, which expands a new research direction of IPR. Finally, using the panel moderation regression models, we reveal the heterogeneous impact of WCDIP on MFP from the perspectives of firm scale, property rights nature, and industry technology level, which complements the literature on the heterogeneity of firm performance research.

The remaining part of this paper is organized as follows: the second part defines the concept of WCDIP, summarizes the literature views related to WCDIP and firm performance, and presents the research hypotheses of this paper; the third part is the empirical research design scheme, providing detailed information about the sample and variable selection; the fourth part is the empirical research results and analysis, including benchmark regressions, model endogeneity problem solving, and robustness tests; finally, we summarize the research findings and point out the practical implications.

## 2. Related concepts and research hypotheses

### 2.1. The whole chain development of intellectual property

In September 2021, the State Council released the “Outline for the Construction of a Strong Intellectual Property Country (2021–2035),” which proposed to open up the whole chain of intellectual property creation, utilization, protection, management, and service, thus clarifying the concept of WCDIP and the path to achieve the construction of a strong IPR Country. There are few researchers on WCDIP in the existing literature, but some scholars have integrated several dimensions of WCDIP based on system principle and synergy theory. McCurdy and Phelps (2002) analyzed the internal composition of the IPR management system based on systematics theory, and regard the IPR management system as an organic composition of intellectual property protection, acquisition, utilization, and licensing subsystems. From a strategic perspective, Al-Aali and Teece (2013) argued that IPR should be managed in an integrated manner, rather than separated according to creation, utilization, and protection. Shan (2014), Chen H. et al. (2022), and Li et al. (2022) integrated the IPR creation subsystem, utilization subsystem, protection subsystem, and management subsystem into one system. Yang et al. (2018) analyzed the speed characteristics of the collaborative evolution of China’s intellectual property development subsystem, operation subsystem, and protection subsystem from the perspective of the evolutionary speed state and evolutionary speed trend, and explored their evolutionary quality and comprehensive validity. Based on the existing theoretical foundation, this study will measure WCDIP in five dimensions: creation, utilization, protection, management, and service, and explore the relationship between WCDIP and MFP.

### 2.2. The whole chain development of intellectual property and manufacturing firm performance

With the development of new growth theories, scholars have focused on the subsequent impacts of IPR development at the macro level (Falvey et al., 2006). On the one hand, it has been argued that these effects are reflected at the macro level, such as a country’s economic growth, innovation, productivity, etc. (Bielig, 2015; Haydaroglu, 2015; Awaworyi Churchill et al., 2022). On the other hand, studies have shown that IPR development also acts at the micro-firm level (Branstetter et al., 2006; Wu and Tang, 2016; Van Stel et al., 2019; Wang and Hu, 2021). This paper will discuss the relationship between WCDIP and MFP from five dimensions: creation, utilization, protection, management, and service. First of all, IPC is the process of developing knowledge products through innovation activities by innovation subjects, and it is the premise of WCDIP. The development of IPC reflects the potential of innovation subjects to generate IPR. IPC can help manufacturing firms maintain their competitive advantage (Cho et al., 2015). This will motivate firms to increase their R&D investment and achieve a high quantity and quality of IPR output, which will improve their performance (Hazarika, 2021; Leung and Sharma, 2021). Second, IPU is the process of commercialization and marketization of IPR achievements, and it is the ultimate purpose of WCDIP. The development of IPU reflects the potential of realizing IPR value by industrializing and capitalizing on IPR achievements. Manufacturing firms tend to use IPR results on their products or license them to others (Cockburn, 2009), both of which generate revenue for the firm (Xu and Yang, 2019). Third, IPP is the process of protecting IPR according to law, and it is a strong guarantee for WCDIP. The development of IPP reflects the attitude and strength of the state in safeguarding the legitimate rights of innovation subjects by formulating and implementing relevant laws and regulations. IPP can reduce the occurrence of infringement, and manufacturing firms will be more inclined to increase R&D investment, consolidate patent reserves, actively participate in market transactions, and strive to improve firm performance through IPR activities (Allred and Park, 2007; Wu and Tang, 2016; Wang and Hu, 2021). In addition, IPM is the process of implementing the legal system of intellectual property through the adoption of a series of management actions by the relevant state departments, and it is the foundation of WCDIP. The development of IPM reflects the state’s level of examination, supervision, coordination, and service on IPR acquisition and utilization. IPM provides firms with management mechanisms and standards that stimulate manufacturing firms to carry out IPR activities more efficiently and thus profitably in the competitive marketplace (Grimaldi et al., 2021; Le Thi et al., 2022). Using a sample of 308 Korean firms, Yong-Hyung and Ki-suk (2016) found that IPM policies have a significant positive impact on the business performance of startups. Finally, IPS is a special service that includes legal service and professional technical services, and it runs through all aspects of WCDIP. The development of IPS reflects the level of providing information, agency, and consultation for innovation subjects through the IPR public service system. As seen in the “Evaluation Report of China’s IPR Development Status in 2020” published in 2021, the number of IPS providers and personnel has increased every year since 2010. By 2020, the number of IPS institutions reached 61,000 and the number of licensed patent attorneys reached 22,000. The IPR pledge financing service index increased by 42.0% from 2019. IPS can facilitate IPC, IPU, and IPM for manufacturing firms, thus contributing to the improvement of their performance. Based on this, the paper puts forward the following research hypotheses:

*Hypothesis 1:* The whole chain development of intellectual property has a promotion effect on manufacturing firm performance.

### 2.3. The whole chain development of intellectual property, firm heterogeneity, and manufacturing firm performance

This paper will explore the heterogeneous impact of WCDIP on MFP from three aspects: firm size, property rights nature, and industry technology level.

According to the quartiles of operating revenue, manufacturing firms can be divided into mega, large, medium, and small firms (Xiong, 2021). Large-scale manufacturing firms have more advantages than small-scale manufacturing firms in facing the opportunities brought by WCDIP. On the one hand, large-scale manufacturing firms have more deployable resources and financing channels (Zhuang et al., 2022), and already have a scale advantage, which enables large-scale manufacturing firms to invest more human and material resources in innovation activities, so they have more intensive IPR activities, pay more attention to the development of external IPR environment, and obtain competitive advantages in IPR development (Zhang and Peng, 2016). Whereas small-scale manufacturing firms have relatively few IPR activities due to the lack of innovation ability, which makes it difficult for them to profit from the development of the external IPR environment. On the other hand, large-scale manufacturing firms have well-developed IPM institutions and management systems, and rich IPM experience, to implement external IPR policies more accurately (González-Álvarez and Nieto-Antolín, 2007). While small-scale manufacturing firms are faced with many constraints in the implementation of IPR policies due to a lack of experience in IPR development (Agostini et al., 2016; Zhuang et al., 2022). Empirical studies have been conducted, which also show this view (Macdonald, 2004; Suh and Hwang, 2010). Based on this, this paper proposes the following research hypothesis:

*Hypothesis 2:* Compared with small-scale manufacturing firms, the whole chain development of intellectual property has a stronger promotion effect on the large-scale manufacturing firm performance.

State-owned manufacturing firms and non-state-owned manufacturing firms have heterogeneity in the relationship between WCDIP and firm performance because of the special institutional background, and the heterogeneity is manifested in political attributes and firm governance. On the one hand, the heavy policy burden may cause state-owned manufacturing firms to violate their business objectives and have a relatively low willingness to engage in innovative activities with high risks and uncertain returns (Tang and Sun, 2014; Duan and Kang, 2022), while non-state-owned manufacturing firms have a lighter policy burden, and are brave enough to engage in technological innovation and IPR activities to achieve high returns, so they rely more on WCDIP. On the other hand, in terms of firm governance, managers of state-owned manufacturing firms are not the actual decision-makers, and there is a mismatch between risk-taking and benefits (Chen L. et al., 2022). The management of state-owned manufacturing firms is doubly constrained in terms of economic and political incentives (Low, 2009; Huang and Li, 2019), which limits the innovative activities of managers. However, managers of non-state-owned manufacturing firms are the actual decision-makers, and their benefits and risks are consistent, coupled with a perfect residual ownership distribution system, which makes managers more willing to innovate, pay more attention to the dynamics of external WCDIP, and more adept in seizing the opportunities brought by WCDIP to improve firm performance (Yuan et al., 2015). Based on this, this paper proposes the following research hypothesis:

*Hypothesis 3:* Compared with state-owned manufacturing firms, the whole chain development of intellectual property has a stronger promotion effect on the non-state-owned manufacturing firm performance.

According to the industry technology level, manufacturing firms can be divided into low-technology (low-tech) manufacturing firms, medium-technology (medium-tech) manufacturing firms, and high-technology (high-tech) manufacturing firms. Studies have shown that medium- and high-technology manufacturing firms have an advantage in technological innovation and IPR development compared to low-technology manufacturing firms (Acemoglu and Akcigit, 2012; Zheng and Song, 2012; Woo et al., 2015). On the one hand, medium- and high-tech industries have higher technology intensity and stronger technological innovation capability (Luo and Liu, 2009). In order to maintain their competitive advantages, medium- and high-tech manufacturing firms tend to increase technological innovation, pay more attention to the IPR environment, and adjust their innovation direction and IPR strategies in time according to changes in the external environment. Whereas low-tech manufacturing firms lack innovation and IPR activities, the promotion effect of WCDIP on firm performance is also limited. On the other hand, a higher technology level means higher barriers to entry and lower competition in the market. In addition, the achievements of high-level IPR are not easy to be imitated, so medium- and high-tech manufacturing firms are more motivated to carry out innovation and IPR activities (Wang and Xu, 2005), which further releases the promotion effect of WCDIP on the medium- and high-tech manufacturing firm performance. Based on this, this paper proposes the following research hypothesis:

*Hypothesis 4:* Compared with low-tech manufacturing firms, the whole chain development of intellectual property has a stronger promotion effect on the medium-and high-tech manufacturing firm performance.

## 3. Research design

### 3.1. Confirmation and measurement of manufacturing firm performance

The explanatory variable in this paper is the level of manufacturing firm performance. The current indicators for measuring the level of firm performance are broadly divided into two categories: first, the indicators based on firm finances represented by the return on total assets and return on net assets; second, the indicators based on capital market returns represented by Tobin’s Q value. Financial indicators can only respond to historical data, but react slowly to changes in firm performance and require a certain response period (Li and Kong, 2005). In this paper, Tobin’s Q value is used to measure MFP based on the following two considerations. First, the sample selected for this study is listed as manufacturing firms, which are more active in terms of market performance. Tobin’s Q value is an indicator to measure the market performance of firms, and the selection of Tobin’s Q value can make the results more accurate. Second, compared with financial indicators, Tobin’s Q value can reflect and predict the current and future value of companies, and passive disclosure will not be subjectively manipulated by management (Hai et al., 2020), so it can more sensitively and objectively reflect the changes of manufacturing firms with the changes of WCDIP, which is more consistent with this study. This study uses Tobin Q to measure MFP in the main effect test, drawing on the research of Swift (2013), and Wu and Xiao (2016). In the robustness test, we use the logarithmic value of operating income (lnOR) to measure MFP, drawing on the research of Liu and Chen (2010). Tobin’s Q value is the ratio of a firm market value to the replacement cost of its assets, which is calculated as follows:

Tobin’s Q value = (price per share × number of tradable shares + net assets per share × number of non-tradable shares + liabilities)/total assets.

### 3.2. Confirmation and measurement of the whole chain development of intellectual property

The core explanatory variable of this paper is WCDIP, which is divided into five dimensions: intellectual property creation (IPC), intellectual property utilization (IPU), intellectual property protection (IPP), intellectual property management (IPM), intellectual property service (IPS) according to the “Outline for Building a Powerful Intellectual Property Country (2021–2035).” Based on the specific elaboration of the five dimensions in the previous article, and drawing on the evaluation report on the development of IPR in China issued by the State Intellectual Property Office and the studies of relevant scholars, this paper selects the most representative indicators for each dimension, and the idea of selecting indicators is as follows: (1) Invention patents with novelty, utility, and inventiveness can best reflect the level of IPC, so the indicators are constructed from the stock and increment of invention patents. (2) The level of IPU is ultimately tested by the market, and the higher the activity of the IPR market and the higher the transaction volume, the higher the level of IPU; (3) IPP aims to protect IPC and IPU, and whether intellectual achievements are effectively protected and whether IPR can be traded in a safe environment is the key to measuring the level of IPP (Wu and Tang, 2016; Zhuang et al., 2022). (4) IPM makes the intellectual property activities of innovation subjects more standardized and efficient, and the construction of IPM rules and regulations and the efficiency of IPM are important indicators of the level of IPM; (5) The more dense the intellectual property service institutions and service personnel in a region, the more developed the local IPS are. Therefore, the number of intellectual property service institutions and service personnel can reflect the level of IPS development. The indicators selected based on the above idea and the indicator weights determined by the entropy value method are shown in Table 1.

**Table 1:** The whole chain development of intellectual property index system.

Primary index	Weight	Secondary index	Weight	Indicator Properties
Intellectual Property Creation (IPC)	0.227	Invention patent authorization (IPC1)	0.097	Positive
		Average ownership of invention patents (IPC2)	0.131	Positive
Intellectual Property Utilization (IPU)	0.125	The number of patent application rights and patent transfers (IPU1)	0.044	Positive
		The number of technology market transaction contracts (IPU2)	0.081	Positive
Intellectual property protection (IPP)	0.329	Intellectual property protection awareness of firms and individuals (IPP1)	0.132	Positive
		Enforcement of intellectual property administrative protection (IPP2)	0.085	Positive
		Degree of intellectual property market standardization (IPP3)	0.112	Positive
Intellectual property management (IPM)	0.142	Efficiency of patent examination by unit examiners (IPM1)	0.095	Positive
		The volume of Intellectual Property Laws and Regulations (IPM2)	0.047	Positive
Intellectual property service (IPS)	0.177	The number of intellectual property service providers (IPS1)	0.080	Positive
		The number of personnel in intellectual property service (IPS2)	0.098	Positive

### 3.3. Confirmation and measurement of control variables

We also control for other firm characteristic variables that are likely to have an impact on MFP. Controlling for the total assets and profitability of the firms, the total assets (Lnasset) and net profits (Lnnp) of the sample firms are logarithmically processed to eliminate the bias caused by outliers; the gearing ratio (Leverage) is calculated by dividing the total liabilities by the total assets; in addition, the sample firms are divided into state-owned and non-state-owned firms, with state-owned firms taking the value of 1 and non-state-owned firms taking the value of 0 to control for the nature of property rights of the firms (SOE); finally, we control for the type of firms (Ind) and determine the industry and industry code in which the sample firms are located in each year according to the classification of manufacturing industries in the National Economic Classification (GB/T4754-2017).

### 3.4. Test model design

To test the impact of WCDIP on MFP, this paper constructs the following benchmark regression model:


(1) $${MFP}_{i,t+1}=\alpha_{0}+\alpha_{1}{\mathrm{WCDIP}}_{i,t}+\gamma CV_{i,t}+\delta_{i}+\zeta_{i}+\varepsilon_{\mathrm{it}}$$

(2) $${MFP}_{i,t+1}=\beta_{0}+\beta_{1}{IPX}_{i,t}+\eta CV_{i,t}+\delta_{i}+\zeta_{i}+\varepsilon_{\mathrm{it}}$$

In model (1) and model (2), i denotes individual manufacturing firm and t denotes time. Considering that macro-level WCDIP has a certain delay effect on MFP, MFP lags by one period, and MFP_i,t + 1_ denotes the performance of firm i in year t + 1. The explanatory variable WCDIP_i,t_ denotes the whole chain development of intellectual property faced by firm i in year t. IPX_i,t_ includes intellectual property creation (IPC_i,t_), intellectual property utilization (IPU_i,t_), intellectual property protection (IPP_i,t_), intellectual property management (IPM_i,t_), and intellectual property service (IPS_i,t_). When taking MFP as the explanatory variable, the individual characteristic variables and other financial indicators at the firm level should be considered. These variables are closely related to MFP and may cause estimation bias if omitted. Therefore, the control variables CV_i,t_ include firm total assets, firm net profit, gearing ratio, firm ownership nature, and firm industry technology level. δ_i_ and ζ_i_ denote individual fixed effects and industry fixed effects, respectively, and ε_it_ denotes the error term.

The existing research literature on firm heterogeneity provides a theoretical basis for this paper to infirm firm heterogeneity factors into the empirical research framework of the impact of WCDIP on MFP. Most scholars believe that firms of different scales, property rights nature, and industry technology levels have significant differences in their ability to adapt to the external environment. Therefore, firm size, property rights nature, and industry technology level may be important influencing factors for WCDIP on MFP. In this paper, we add dummy variables such as firm size, property rights nature, and industry technology level to the above benchmark model.


(3) $$\begin{array}{l} {MFP}_{i,t+1}=a_{0}+a_{1}{\mathrm{WCDIP}}_{i,t}+a_{2}{\mathrm{size}}_{i,t,r}+\\ \;a_{3}{{\mathrm{WCDIP}}_{i,t}}^{*}{\mathrm{size}}_{i,t,r}+{aCV}_{i,t}+\delta_{i}+\zeta_{i}+\varepsilon_{\mathrm{it}} \end{array}$$

(4) $$\begin{array}{l} {MFP}_{i,t+1}=a_{0}+a_{1}{\mathrm{WCDIP}}_{i,t}+a_{2}\mathrm{capital}\_{\mathrm{type}}_{i,t}+\\ \;a_{3}{{\mathrm{WCDIP}}_{i,t}}^{*}\mathrm{capital}\_{\mathrm{type}}_{i,t}+{aCV}_{i,t}+\delta_{i}+\zeta_{i}+\varepsilon_{\mathrm{it}} \end{array}$$

(5) $$\begin{array}{l} {MFP}_{i,t+1}=a_{0}+a_{1}{\mathrm{WCDIP}}_{i,t}+a_{2}{\mathrm{industry}}_{i,t}+\\ \;a_{3}{{\mathrm{WCDIP}}_{i,t}}^{*}{\mathrm{industry}}_{i,t+}\;{aCV}_{i,t}+\delta_{i}+\zeta_{i}+\varepsilon_{\mathrm{it}} \end{array}$$

Among them, in the model (3), size_i,t,r_ denotes the dummy variable of firm size, and this paper adopts firm operating revenue as the proxy variable of firm size and divides manufacturing firms into four types according to the quartiles of operating revenue: mega (r takes the value of 1), large (r takes the value of 2), medium (r takes the value of 3), and small (r takes the value of 4). In order to explore the heterogeneous effect of WCDIP on MFP of different sizes, an interaction term between WCDIP and the dummy variable of firm size (WCDIP_i,t_*size_i,t,r_) is added to the model. a_3_ is the coefficient of interactive items. Taking mega-scale firms as a reference, If a_3_ is positive, it indicates that WCDIP has a stronger promotion effect on the performance of small, medium, and large manufacturing firms. In model (4), capital_type_i,t_ is the dummy variable of whether state-owned manufacturing firms. If firm i is a state-owned manufacturing firm in year t, the value is 1; for non-state-owned manufacturing firms, the value is 0. In order to explore the heterogeneous effect of WCDIP on MFP with different property rights, the interaction term between WCDIP and the dummy variable of whether or not a manufacturing firm is state-owned (WCDIP_i,t_*capital_type_i,t_) is added to the model. If a_3_ is significantly positive, it indicates a stronger promotion effect of WCDIP on the performance of state-owned manufacturing firms. In model (5), industry_i,t_ represents the dummy variable of the technology level of the industry in which the firm is located and takes the value of 1 if firm i belongs to the high-technology industry in year t, 2 if it belongs to the medium-technology industry, and 3 if it belongs to the low-technology industry. In order to explore the heterogeneity effect of WCDIP on MFP of different industry technology levels, the interaction term between WCDIP and the dummy variable of the industry technology level (WCDIP_i,t_*industry_i,t_) is added to the model. Taking high-tech manufacturing firms as the reference, if a_3_ is positive, it indicates that WCDIP has a stronger promotion effect on the performance of medium- and low-tech manufacturing firms. The remaining symbols and variables in models (3)–(5) denote the same meanings as the model (1).

### 3.5. Research samples and data sources

This study takes all listed manufacturing firms from 2010 to 2020 as the initial sample, excluding the samples of manufacturing firms in only a few years from 2010 to 2020, as well as firms with delisting and incomplete data, and finally obtains 10,670 observation values of 970 firms. The financial data of listed firms were obtained from CSMAR and public financial statements of listed firms. The data of WCDIP were obtained from the State Intellectual Property Office, the World Intellectual Property Organization (WIPO), Peking University Magic Weapon, the China Science and Technology Statistical Yearbook, and the National Bureau of Statistics. This paper matches the external WCDIP indicators and five sub-dimensional indicators faced by the sample companies according to the year in which they are located. The data of control variables were obtained from the CSMAR database and the firm search database. The missing values of sample data were processed by linear interpolation. In order to eliminate the deviation of the analysis results caused by abnormal values, this study takes logarithmic treatment for the total index.

## 4. Analysis of empirical results

This paper uses Stata 17.0 software to regress the panel data, and the LM test and Hausman test results indicate that the fixed-effects model is better than the mixed-effects model and the random-effects model, so this paper mainly uses the fixed-effects model to carry out the empirical study.

### 4.1. Descriptive statistics

Table 2 reports the descriptive statistics of the variables in this study. The MFP mean value is 2.384 and the standard deviation is 0.689, indicating that MFP has fluctuated greatly in the past 10 years. The maximum value is 3.434 and the minimum value is 0, indicating that the manufacturing industry is generally profitable and there are large differences in performance among firms. The WCDIP mean value is 0.113 and the standard deviation is 0.023, indicating that the level of WCDIP has been stable in the past 10 years, with a slow upward trend, as well as the five sub-dimensions of WCDIP. The correlation test results show that the correlations between the main explanatory variables are not significant, and the correlation coefficients of WCDIP and IPC, IPU, IPM, and IPS with MFP are significantly positive, while the correlation coefficient of IPP with MFP is significantly negative. Therefore, it can be tentatively judged that WCDIP and its four dimensions have a positive effect on MFP. In addition, the variance inflation factor (VIF) is 1.05, which is much smaller than 10, so the multicollinearity problem can be ignored.

**Table 2:** Descriptive statistics of variables.

Variables	*N*	Mean	SD	Min	Max
MFP	10,670	2.384	0.689	0.000	3.434
WCDIP	10,670	0.113	0.023	0.060	0.151
IPC	10,670	0.109	0.037	0.046	0.171
IPU	10,670	0.065	0.032	0.000	0.125
IPP	10,670	0.159	0.024	0.123	0.208
IPM	10,670	0.078	0.033	0.004	0.137
IPS	10,670	0.094	0.057	0.000	0.178
Lnasset	10,670	22.198	1.282	0.000	27.547
Leverage	10,670	0.469	1.782	0.000	178.346
Lnnp	10,670	0.007	0.243	−24.802	0.723
LnOR	10,670	21.636	1.466	13.818	27.512
SOE	10,670	0.421	0.494	0.000	1.000

### 4.2. Main effect test and endogeneity test

Hypothesis 1 suggests that there is a positive relationship between WCDIP and MFP. Columns (1)–(2) of Table 3 display the impact of WCDIP on MFP. Column (1) only controls for individual and industry fixed effects, while column (2) incorporates all control variables. The coefficient estimates for WCDIP are 18.121 and 15.159, respectively, and are significant at the 1% significance level, which indicates that WCDIP has a promotion effect on MFP under the given other variables, thus verifying Hypothesis 1. While most of the existing literature studies individual dimensions of IPC, IPU, IPP, IPM, and IPS, this paper expands on the research related to IPR by examining a comprehensive dimension. In addition, this paper further explores the relationship between the development of IPR sub-dimensions and MFP. Column (3) reports the impact of the five dimensions of IPC, IPU, IPP, IPM, and IPS on MFP. Given the other variables unchanged, IPC, IPU, and IPS have a promotion effect on MFP, and the promotion effect of IPS is the most significant (B_IPS_ = 5.020, *p* < 0.01), followed by IPU (B_IPU_ = 2.594, *p* < 0.01) and IPC (B_IPC_ = 1.228, *p* < 0.01). The IPS promotes MFP by building service platforms and improving service efficiency. The IPU promotes MFP by stimulating market vitality and expanding IPR trading platforms. The IPC promotes MFP by correctly guiding the R&D orientation of firms and consolidating the high-level IPR stock of firms. However, IPP (B_IPP_ = −0.903, *p* < 0.01) and IPM (B_IPM_ = −2.585, *p* < 0.01) have a suppressive effect on MFP, probably because the measures of these two dimensions are more macroscopic than the other three dimensions and involve the promulgation and implementation of policies and legal documents, but the impact of policies and legal documents on micro-firm performance will take some time to reflect. Therefore, this paper takes Tobin’s Q value of t + 2 and t + 3 as explanatory variables to test again. Columns (4)–(5) show that IPP still has a suppressive effect on MFP, probably because China is still at the stage of building an innovative country, with a strong capacity for imitative innovation and a weak capacity for original innovation (van Stel et al., 2019), and an excessive level of IPP will increase the cost of imitative innovation of manufacturing firms and thus negatively affect MFP (Falvey et al., 2006; Gold et al., 2019). IPM has a significant promotion effect on MFP, probably because IPM makes the innovation and IPR activities of manufacturing firms more standardized and efficient, and then promotes MFP. But the promotion effect can only be seen after a certain period due to the lagging effect of policies and regulations.

**Table 3:** The impact of WCDIP on MFP and endogeneity test.

Variables	(1)	(2)	(3)	(4)	(5)	(6)
	L.MFP	L.MFP	L.MFP	L2.MFP	L3.MFP	L.MFP
WCDIP	18.121***	15.159***				
	(106.850)	(80.275)				
UKIP						562.885***
						(5.887)
IPC			1.228***			
			(10.541)			
IPU			2.594***			
			(5.309)			
IPP			−0.903***	−0.117	−2.980***	
			(−5.399)	(−0.623)	(−16.951)	
IPM			−2.585***	4.932***	4.630***	
			(−9.533)	(25.195)	(25.845)	
IPS			5.020***			
			(21.829)			
Lnasset		0.197***	0.083***	0.378***	0.274***	−0.142***
		(30.448)	(14.069)	(44.697)	(29.489)	(−2.747)
Leverage		0.006***	0.005***	0.011***	0.008***	0.073
		(3.496)	(3.168)	(5.113)	(4.233)	(0.893)
Lnnp		−0.013	0.001	−0.023*	−0.017	0.045***
		(−1.198)	(0.144)	(−1.711)	(−1.375)	(3.115)
SOE		0.123***	0.162***	0.079***	0.150***	0.249***
		(5.611)	(8.571)	(2.679)	(5.013)	(4.936)
Idf	Yes	Yes	Yes	Yes	Yes	Yes
Ind	Yes	Yes	Yes	Yes	Yes	Yes
w-R^2^	0.530	0.577	0.688	0.321	0.390	
*N*	9,700	9,700	9,700	8,730	7,760	5,820

Idf and Ind are individual fixed effects and industry fixed effects, respectively; w-R^2^ is the between-group goodness-of-fit; *N* is the sample size; ***, **, * denote significance at 1%, 5%, and 10%, respectively.

Although this paper controls for industry fixed effects, individual fixed effects, and other firm characteristics that affect performance in the baseline regression model, there may still be uncontrollable factors, such as the macroeconomic environment and socio-cultural environment, that may have an impact on WCDIP and MFP, which may lead to some bias in the estimated coefficients. In order to solve the possible endogenous problems, this paper will use the instrumental variables method to further test the accuracy of the research results. Referring to the study of Xiong (2021), this paper takes the WCDIP of the United Kingdom index as a tool variable, because the United Kingdom has frequent trade with China, and the WCDIP of the United Kingdom is correlated with the WCDIP of China, while it is not related to the market performance of China’s manufacturing firms. Therefore, the WCDIP of the United Kingdom index is used as the instrumental variable for endogeneity analysis. If the level of IPR development in the United Kingdom is higher than that in China in that year, the value is 1, otherwise, the value is 0. Considering the correlation between instrumental variables and endogenous variables, this paper uses three kinds of statistics to test. First, the Kleibergen-Paap rk LM statistic is used to test the hypothesis of “under-identification of instrumental variables,” which is rejected at the 1% level. Second, the Kleibergen-Paap rk Wald F-statistic is 30.388, which is higher than the 10% level critical value of the Stock-Yogo test, and the Cragg-Donald Wald F-statistic is 30.573, which is higher than the 10% level critical value of Stock-Yogo test, all of which reject the hypothesis of weak identification of instrumental variables. Finally, the value of the first-stage F-statistic is 34.8, which is much higher than 10, so it can be concluded that there is no weak instrumental variable problem. In summary, it can be judged that there is a strong correlation between instrumental variables and endogenous variables. Therefore, the instrumental variables selected in this paper satisfy the application conditions. The results of the first-stage regression show that the coefficient of the instrumental variables is significantly positive at the 1% significance level, which indicates that there is a significant positive relationship between the level of WCDIP in China and the level of IPR development in China and the United Kingdom. The estimation results of the two-stage least squares method reported in column (6) show that although the coefficients estimated by the instrumental variables method are somewhat different from the coefficients of the benchmark regression, the signs and significance of the core variables are consistent with the results of the benchmark regression, so the results of the benchmark regression are relatively robust and reliable.

### 4.3. Heterogeneity effect test

This paper further explores the heterogeneous effects of WCDIP on MFP in terms of size differences,^1^ property rights nature differences, and technology level differences.^2^

Hypothesis 2 suggests that WCDIP is more beneficial to the performance of large-scale manufacturing firms. Columns (1)–(4) of Table 4, respectively, report the impact of WCDIP on MFP of different sizes. The results show that WCDIP has a significant positive effect on MFP of all sizes, and the influence degree from strong to weak is small-, medium-, large-, and mega-scale firms. Column (5) shows that when the interaction term WCDIP_i,t_*size_i,t,r_ coefficient is based on mega-scale firms, WCDIP has a stronger promotion effect on the large- and medium-scale manufacturing firm performance, thus rejecting Hypothesis 2. This may be because small-scale manufacturing firms are more sensitive and flexible in decision-making, and are more able to quickly detect and adapt to WCDIP, and improve their performance. In contrast, large-scale manufacturing firms often have mature development, complex organizational structure, and a set of standardized operation modes, which makes it difficult for them to respond quickly to the external environment (Tripsas and Gavetti, 2017). In addition, relevant studies support this conclusion (Arora and Fosfuri, 2003; Motohashi, 2008). Hypothesis 3 suggests that WCDIP is more beneficial to the performance of non-state manufacturing firms. Columns (6)–(7) report the impact of WCDIP on MFP with different property rights, and the results show that WCDIP has a significant promotion effect on the performance of both state-owned and non-state-owned manufacturing firms, especially at a higher level for non-state-owned manufacturing firms, thus verifying Hypothesis 3. Column (8) shows that the interaction term WCDIP_i,t_*capital_type_i,t_ coefficient is significantly negative at the 1% level, which indicates that WCDIP has a stronger promotion effect on the non-state manufacturing firm performance, again verifying Hypothesis 3. This may be because the heavy policy burden and imperfect incentive system limit the motivation of state-owned manufacturing firms in innovation activities, and non-state-owned manufacturing firms can seize the opportunity to develop rapidly (Huang and Li, 2019). Hypothesis 4 suggests that WCDIP is more beneficial to the performance of medium- and high-technology manufacturing firms. Columns (9)–(11) report the impact of WCDIP on MFP at different technology levels, and the results show that WCDIP has a significant promotion effect on the performance of high-, medium-, and low-tech manufacturing firms, especially on medium-tech manufacturing firms at a higher level, followed by high-and low-tech manufacturing firms, thus verifying Hypothesis 4. Column (12) shows that when the interaction term WCDIP_i,t_*industry_i,t_ coefficient is based on high-tech manufacturing firms, WCDIP has a stronger promotion effect on the medium-tech manufacturing firm performance, but has no significant effect on the low-tech manufacturing firm performance. This indicates that high-tech manufacturing firms are more dependent on innovation and have a higher patent density, and can seize the opportunities and convenience brought by WCDIP in time to develop IPR, thus promoting firm performance. This is consistent with the conclusions reached by scholars (Acemoglu and Akcigit, 2012; Woo et al., 2015).

**Table 4:** The heterogeneity impact of WCDIP on MFP.

Variables	(1)	(2)	(3)	(4)	(5)	(6)	Firm scale	Interaction term	Property rights nature	Small-scale	Medium-scale	Large-scale	Mega-scale		State-owned	L.MFP	L.MFP	L.MFP	L.MFP	L.MFP	L.MFP
WCDIP	21.011***	14.562***	10.788***	7.614***	14.957***	10.245***
	(49.085)	(35.244)	(33.165)	(29.543)	(71.103)	(52.309)
WCDIP*size_2_					0.575***	
					(5.337)	
WCDIP*size_3_					0.656***	
					(4.635)	
WCDIP*size_4_					−0.259	
					(−1.412)	
WCDIP*capital_type						
						
WCDIP*industry_2_						
						
WCDIP*industry_3_						
						
Lnasset	0.211***	0.328***	0.254***	0.251***	0.198***	0.130***
	(11.053)	(14.613)	(14.078)	(22.481)	(24.749)	(16.930)
Leverage	0.004**	0.144**	0.122**	−0.165***	0.006***	−0.065**
	(2.156)	(2.397)	(2.471)	(−3.529)	(3.644)	(−2.354)
Lnnp	−4.213***	0.062	−0.335	−0.025***	−0.012	−0.013*
	(−4.526)	(0.078)	(−0.760)	(−3.471)	(−1.120)	(−1.651)
SOE	0.081*	0.100**	−0.032	0.066	0.116***	0.000
	(1.699)	(2.367)	(−0.709)	(1.568)	(5.332)	
Idf	Yes	Yes	Yes	Yes	Yes	Yes
Ind	Yes	Yes	Yes	Yes	Yes	Yes
w-R^2^	0.580	0.492	0.428	0.559	0.582	0.5659
*N*	2,282	2,418	2,477	2,523	9,700	4,058
**Variables**	**(7)**	**(8)**	**(9)**	**(10)**	**(11)**	**(12)**
	**Property rights nature**	**Interaction term**	**Technology level**			**Interaction term**
	**Non-state-owned**		**High-tech**	**Medium-tech**	**Low-tech**	
	**L.MFP**	**L.MFP**	**L.MFP**	**L.MFP**	**L.MFP**	**L.MFP**
WCDIP	18.642***	19.530***	14.805***	15.598***	13.941***	14.978***
	(65.811)	(85.373)	(35.359)	(64.583)	(31.181)	(61.447)
WCDIP*size_2_						
						
WCDIP*size_3_						
						
WCDIP*size_4_						
						
**Variables**	**(7)**	**(8)**	**(9)**	**(10)**	**(11)**	**(12)**
	**Property rights nature**	**Interaction term**	**Technology level**			**Interaction term**
	**Non-state-owned**		**High-tech**	**Medium-tech**	**Low-tech**	
	**L.MFP**	**L.MFP**	**L.MFP**	**L.MFP**	**L.MFP**	**L.MFP**
WCDIP*capital_type		−9.637***				
		(−30.776)				
WCDIP*industry_2_						0.461**
						(2.387)
WCDIP*industry_3_						−0.154
						(−0.482)
Lnasset	0.234***	0.180***	0.209***	0.199***	0.228***	0.194***
	(24.876)	(29.282)	(15.276)	(22.963)	(11.365)	(30.127)
Leverage	0.006***	0.004***	0.062***	0.005**	0.163***	0.005***
	(3.047)	(2.647)	(4.715)	(2.573)	(2.746)	(3.290)
Lnnp	−0.898***	−0.013	−0.176	−0.012	−1.003***	−0.013
	(−4.166)	(−1.215)	(−0.731)	(−1.060)	(−2.989)	(−1.184)
SOE	0.000	1.261***	0.179***	0.065**	0.103**	0.126***
		(29.713)	(4.048)	(2.120)	(2.099)	(5.742)
Idf	Yes	Yes	Yes	Yes	Yes	Yes
Ind	Yes	Yes	Yes	Yes	Yes	Yes
w-R^2^	0.632	0.618	0.569	0.570	0.576	0.572
*N*	5,642	9,700	2,450	5,793	1,457	9,700

### 4.4. Robustness testing

In order to further verify the robustness of the research results, this paper conducts robustness tests by re-screening the sample and replacing the explanatory variables. First, considering that 2018 is the most prominent year in terms of the level of WCDIP^3^, the reliability of the sample data may have some bias, so the sample data of 2018 is excluded. Column (1) of Table 5 shows that there is still a positive and significant effect of WCDIP on MFP after screening the sample. Second, this paper replaces Tobin’s Q value with lnOR to characterize the MFP based on the screening sample. Columns (2)–(3) show that there is still a positive and significant effect of WCDIP on MFP after replacing the explanatory variables. In summary, it can be seen that the results of the main effect have strong robustness. Table 6 shows the results of the robustness test for the heterogeneity effect based on the screening sample, where columns (1)–(4), (5)–(6), and (7)–(9) show the robustness test results for the heterogeneity effect of firm size, property rights nature, and industry technology level. The results indicate that WCDIP still has a significant heterogeneity effect on MFP after screening the sample.

**Table 5:** Robustness test of the impact of WCDIP on MFP.

Variables	(1)	(2)	(3)
	L.TQ (Delete 2018)	L.lnOR	L.lnOR (Delete 2018)
WCDIP	15.671***	2.210***	2.798***
	(85.771)	(8.223)	(10.187)
Lnasset	0.125***	0.750***	0.684***
	(17.325)	(81.562)	(63.256)
Leverage	0.004***	0.001	−0.001
	(2.621)	(0.425)	(−0.265)
Lnnp	−0.449***	−0.052***	0.142
	(−3.196)	(−3.277)	(0.672)
SOE	0.129***	0.100***	0.109***
	(5.467)	(3.205)	(3.052)
Idf	Yes	Yes	Yes
Ind	Yes	Yes	Yes
w-R^2^	0.632	0.507	0.469
*N*	7,760	9,700	7,760

**Table 6:** Robustness test of the heterogeneity impact of WCDIP on MFP.

Variables	(1)	(2)	(3)	(4)	(5)	(6)	(7)	(8)	(9)
	Firm scale				Property rights nature		Technology level		
	Small-scale	Medium-scale	Large-scale	Mega-scale	State-owned	Non-state-owned	High-tech	Medium-tech	Low-tech
	L.MFP	L.MFP	L.MFP	L.MFP	L.MFP	L.MFP	L.MFP	L.MFP	L.MFP
WCDIP	20.660***	16.387***	12.826***	9.592***	10.839***	19.695***	16.275***	15.973***	14.560***
	(51.059)	(39.710)	(38.743)	(35.494)	(59.264)	(72.423)	(38.754)	(68.804)	(34.292)
Lnasset	0.198***	0.193***	0.136***	0.124***	0.058***	0.131***	0.102***	0.132***	0.138***
	(9.901)	(8.052)	(6.707)	(8.941)	(7.027)	(12.779)	(6.623)	(13.750)	(6.394)
Leverage	0.005**	0.314***	0.253***	0.015	0.084***	0.004**	0.058***	0.004**	0.344***
	(2.446)	(5.002)	(4.886)	(0.287)	(2.943)	(2.169)	(4.609)	(2.022)	(5.458)
Lnnp	−7.074***	2.091**	1.615**	0.016	0.095	−0.357	0.092	−0.525***	−1.226***
	(−2.779)	(2.379)	(2.552)	(0.139)	(0.772)	(−1.577)	(0.374)	(−2.755)	(−3.137)
SOE	0.143***	0.087*	0.031	0.026	0.000	0.000	0.155***	0.087***	0.124**
	(2.954)	(1.872)	(0.614)	(0.603)			(3.172)	(2.619)	(2.381)
Idf	Yes	Yes	Yes	Yes	Yes	Yes	Yes	Yes	Yes
Ind	Yes	Yes	Yes	Yes	Yes	Yes	Yes	Yes	Yes
w-R^2^	0.639	0.568	0.549	0.637	0.641	0.695	0.619	0.631	0.640
*N*	1,968	1,995	1,910	1,887	3,253	4,507	1,948	4,637	1,175

## 5. Discussion

H1 confirms the positive association between WCDIP and MFP, and the prior literature supports our findings. This implies that the better the macro WCDIP environment is, the more favorable it is for MFP. The specific mechanism of the effect of WCDIP on MFP can be reflected in five dimensions: IPC, IPU, IPP, IPM, and IPS. IPC development can stimulate manufacturing firms to increase R&D investment and innovation output, and studies have shown that patent application can improve the innovation performance of firms and have a positive impact on firm performance (Andries and Faems, 2013; Cho et al., 2015; Leung and Sharma, 2021). IPU development provides a good market trading environment for firms, which provides a good basis for manufacturing firms to transform and transfer IPR results. The existing literature suggests that firms tend to transfer patents to external parties (Cockburn, 2009), which brings additional revenue to firms (Gans et al., 2002). IPP development can help firms to defend their legal rights and reduce external infringements, thus increasing their incentive to engage in IPR activities and profit from them. While this has been demonstrated in relevant studies, it has also been suggested that IPP does not promote performance growth, which is consistent with the results of the empirical tests in this paper. This may also be related to the current stage of China as a developing country (Falvey et al., 2006; Gold et al., 2019). IPM development enhances the competitiveness of firms by setting development standards and standardizing the IPM activities of manufacturing firms. There are empirical studies that support this view (Yong-hyung and Ki-suk, 2016), but the implementation of IPM standards requires a certain period to work on firm performance. IPS development helps firms to quickly access IPR-related information and facilitates efficient IPR activities, which will benefit firms to improve their performance.

H2 shows that WCDIP is more beneficial to the performance of large-scale manufacturing firms compared to small-scale manufacturing firms. Scholars have explained this in terms of accessible resources and financing channels, suggesting that large-scale firms not only have more human, financial, and material resources but also more channels for financing, which facilitates large-scale firms to quickly identify opportunities in the external environment of IPR development and gain competitive advantages from them (Zhang and Peng, 2016; Zhuang et al., 2022). It has also been shown that stronger IPR conditions stimulate large-scale firms to increase R&D expenditures and boost sales revenue. In contrast, small-scale firms will not be able to generate better sales revenue due to budget constraints (Cho et al., 2015). This is contrary to the empirical test results in this paper, which may be because small-scale manufacturing firms are more responsive and quicker to make decisions, adapt quickly to changes in the external environment, and take the lead to improve their performance, while large-scale manufacturing firms are slower to respond to changes in the external environment due to organizational inertia.

H3 shows that WCDIP is more beneficial to the performance of non-state manufacturing firms compared to state-owned manufacturing firms. The results of this paper confirm this hypothesis, which has also been verified in many studies. It shows that non-state manufacturing firms have the advantages of lighter policy burden and better incentive system than state-owned manufacturing firms, and managers of non-state manufacturing firms are more willing to innovate and carry out IPR activities, so non-state manufacturing firms are better at finding opportunities to develop themselves in WCDIP environment (Low, 2009; Tang and Sun, 2014; Huang and Li, 2019; Duan and Kang, 2022).

H4 indicates that WCDIP is more beneficial to the performance of medium- and high-technology manufacturing firms compared to low-tech manufacturing firms. Compared to low-tech manufacturing firms, medium- and high-tech manufacturing firms are more dependent on innovation, more motivated to conduct R&D, and able to seize the opportunities brought by macro WCDIP promptly to actively develop IPR, thus improving firm performance. Previous studies have also found the same findings and support our study (Wang and Xu, 2005; Cho et al., 2015).

All along, China attaches great importance to the development of the manufacturing industry and regards WCDIP as a driving force for the high-quality development of the manufacturing industry. WCDIP refers to the synergistic development of the five dimensions of creation, utilization, protection, management, and service, rather than the single-dimensional development. In the existing studies, few scholars have explored the relationship between WCDIP and firm performance from a comprehensive perspective, and most scholars have only studied its effect on firm performance from a certain dimension. For example, scholars such as Wu and Tang (2016) and Wang and Hu (2021) found that IPP had a significant contribution to firm performance. As a comprehensive dimension, there may also be a link between WCDIP and MFP. In addition, in corporate practice, firms set up IPR affairs departments to quickly adapt to changes in the external IPR environment, which are specifically responsible for IPR work. It is evident that firms attach importance to the macro IPR development environment. The research findings of this paper have certain reference values for the government and manufacturing firms. On the one hand, it provides direction for the government to optimize the strategic layout of IPR. The government should formulate specific policies according to the characteristics of different types of firms to maximize the effectiveness of the policies. On the other hand, manufacturing firms should strengthen their internal IPR management to be able to seize opportunities in the IPR development environment.

## 6. Research conclusions and implications

### 6.1. Research conclusions

The results of this empirical study show that WCDIP can significantly promote MFP, but the five dimensions of IPC, IPU, IPP, IPM, and IPS have different effects on MFP. IPC, IPU, and IPS have a significant promotion effect on MFP, among which IPS has the strongest promotion effect, followed by IPU and IPC. Professional IPS improves the efficiency and effectiveness of intellectual property activities of manufacturing firms, which, in turn, promotes manufacturing firm performance; IPU helps manufacturing firms to realize the transformation of intellectual property achievements by stimulating intellectual property market activity, which, in turn, promotes MFP; IPC maintains the sustainable competitive advantage of manufacturing firms by correctly guiding the direction of R&D and increasing the stock of creative achievements, which, in turn, promotes MFP. There is a significant negative relationship between IPP, IPM, and MFP. Considering that IPM involves the promulgation and implementation of policies and regulations, and the effect of policies and regulations has a lag, this paper tests the firm performance again with a lag of three periods. The results show that IPM has a significant promotion effect on MFP, while IPP and MFP are still significantly negatively correlated. IPM makes the innovation and intellectual property activities of manufacturing firms more standardized and efficient, and thus promotes MFP, but the lagging effect of policies and regulations makes this promotion effect appear only after a certain period. IPP always has a suppressive effect on MFP, probably because China is still in the construction stage of an innovative country, with strong imitation innovation capability and insufficient original innovation capability, and the excessive level of IPP will increase the cost of imitation innovation of manufacturing firms and thus negatively affect MFP.

In addition, the promotion effect of WCDIP on MFP of different types is heterogeneous. In terms of firm size, the promotion effect of WCDIP on MFP is small, medium, large, and mega manufacturing firms from strong to weak. In terms of property rights nature, the promotion effect of WCDIP on the performance of non-state manufacturing firms is significantly stronger than that of state-owned manufacturing firms. In terms of industry technology level, WCDIP has the strongest promotion effect on the performance of medium-tech manufacturing firms, followed by high-tech manufacturing firms, and finally low-tech manufacturing firms. Furthermore, the results of the endogeneity test based on the instrumental variables method and the robustness test based on re-screening the sample and replacing the explanatory variables also support the above findings.

### 6.2. Research implications

Based on the above research findings, this paper will put forward policy recommendations from both government and firm levels.

This paper suggests that the government authorities further optimize the strategic layout of the whole chain development of intellectual property, and actively promote the performance of all types of manufacturing firms by combining targeted policies with complementary policies. Specific policy recommendations are as follows: ① Implement the goal of building a strong intellectual property country, strengthen the coupling and synergy of the five dimensions of IPC, IPU, IPP, IPM, and IPS, realize the benign interaction between the top-level design of WCDIP and the bottom-level logic of manufacturing firms, and build an institutional mechanism to stimulate the innovation and intellectual property development vitality of manufacturing firms; Exert the advantages of strong promotion of IPS, and build an intellectual property public service system of “government guidance, market drive, firm participation and platform sharing” around the needs of manufacturing firms; Improve the level of intellectual property creation and cultivate high-value patents in frontier fields and key areas in multiple directions; Promote IPU, establish an open and transparent intellectual property trading platform, and stimulate the vitality of intellectual property operations of manufacturing firms; Realize the modernization of intellectual property management system and establish an intellectual property management system with static management as the mainstay and dynamic management as the supplement; Moderately stimulate the development potential of IPP, build an intellectual property protection system with “complete structure, fairness and transparency, diversified participation and smooth connection,” and achieve the optimal level of IPP. ② Appropriately broaden and increase the breadth and intensity of government subsidies for small- and medium-scale manufacturing firms. Further, break the institutional barriers and resource bottleneck of non-state manufacturing firms in innovation development. Actively exert the innovation leading role of medium- and high-tech manufacturing firms, promote the organic integration of the innovation value chain of high-, medium- and low-tech manufacturing firms, and fully release the strong promotion effect of WCDIP on the performance of small-scale, non-state, medium- and high-tech manufacturing firms. ③ To improve the performance of large-scale, state-owned, low-tech manufacturing firms, it is not enough to rely on WCDIP alone, and diversified policies are needed. For example, guide large-scale manufacturing firms to high-end, intelligent, and green development by implementing intelligent manufacturing engineering and digital transformation, and realize precise service for large-scale manufacturing firms by establishing the “firm through train” system. Promote the innovation in the operational and mechanism of mixed ownership reform of state-owned manufacturing firms, and enhance the environmental insight and response sensitivity of state-owned manufacturing firms. Strengthen the awareness of transformation and upgrading of low-tech manufacturing firms, increase the assistance during the transition period of transformation and upgrading, and enhance the status of low-tech manufacturing firms in the international division of labor.

In addition, manufacturing firms should respond positively to the national WCDIP policy, solve the problem of unbalanced and insufficient IPR development, and cooperate to promote the high-quality development of the manufacturing industry. The specific policy suggestions are as follows: ① Manufacturing firms should implant the concept of IPR development, actively study and judge the national WCDIP policy, grasp the policy dividends to strengthen the foundation of innovation and IPR development, and build the management mechanism of synergy between IPR and firm performance. ② Small-scale, non-state-owned, medium- and high-tech manufacturing firms should build a framework system of high-quality output, rapid transformation, key protection, and standard management of IPR as soon as possible, make full use of the strong driving effect of WCDIP on performance, and enhance the core competitiveness and international competitiveness of IPR. ③ Large-scale, state-owned, low-tech manufacturing firms should overcome their obstacles such as low efficiency, rigid organization, and backward consciousness, tap the shortcomings of insufficient innovation and IPR development and apply precise measures and targeted efforts to collaboratively promote the manufacturing industry out of the dilemma of being large but not strong.

## Data availability statement

The raw data supporting the conclusions of this article will be made available by the authors, without undue reservation.

## Author contributions

LY design of the study, the conception of the whole chain development of intellectual property, writing, review, and editing. CZ acquisition of the data and analysis, writing—original draft preparation, organizing revisions, and revising manuscript format. All authors contributed to the article and approved the submitted version.

## Funding

This work was supported by the National Social Science Fund Youth Project “Research on Intellectual Property Capability System of Patent-intensive firms in China” (project no.: 16CGL010) and the Basic Scientific Research Business Expenses Project of Heilongjiang Provincial Universities “Research on Intellectual Property Capability Evaluation of firms from the Perspective of Operation Process” (project no.: 2020-KYYWF-0924).

## Conflict of interest

The authors declare that the research was conducted in the absence of any commercial or financial relationships that could be construed as a potential conflict of interest.

## Publisher’s note

All claims expressed in this article are solely those of the authors and do not necessarily represent those of their affiliated organizations, or those of the publisher, the editors and the reviewers. Any product that may be evaluated in this article, or claim that may be made by its manufacturer, is not guaranteed or endorsed by the publisher.

## Footnotes

^1^The quartiles of operating income of listed manufacturing firms are used to determine the size types of firms, and the ranges of four types of firms are calculated separately, including mega scale (above ¥589,022,000), large scale (¥224,229–589,022,000), medium scale (¥957,650–224,229,000) and small scale (below ¥957,650,000).

^2^The OECD International Standard Industrial Classification, Version 3, is used to determine the technology level of the industry to which manufacturing firms belong, with high technology industries including manufacturing industries coded as 27, 37, 39, and 40, medium technology industries including manufacturing industries coded as 38, 36, 28, 26, 37, 34, 35, 29, 30, 31, 32, 33, 25, 43, and 42, and low technology industries including manufacturing industries coded as 13, 14, 15, 16, 17, 18, 19, 20, 21, 22, 23, 24, and 41.

^3^In the "Evaluation Report of China’s IPR Development Status in 2020," by making an annual comparison of the comprehensive IPR development index from 2010 to 2020, we find a year-on-year growth rate of 17.9% for the comprehensive IPR development index in 2018, compared to other years when the year-on-year growth rate is below 10%.
